# Methadone Maintenance Treatment Promotes Referral and Uptake of HIV Testing and Counselling Services amongst Drug Users and Their Partners

**DOI:** 10.1371/journal.pone.0152804

**Published:** 2016-04-05

**Authors:** Bach Xuan Tran, Long Hoang Nguyen, Lan Phuong Nguyen, Cuong Tat Nguyen, Huong Thi Thu Phan, Carl A. Latkin

**Affiliations:** 1 Institute for Preventive Medicine and Public Health, Hanoi Medical University, Hanoi, Vietnam; 2 Johns Hopkins Bloomberg School of Public Health, Baltimore, Maryland, United States of America; 3 School of Medicine and Pharmacy, Vietnam National University, Hanoi, Vietnam; 4 Harvard T.H Chan School of Public Health, Boston, Massachusetts, United States of America; 5 Institute for Global Health Innovations, Duy Tan University, Da Nang, Vietnam; 6 Authority of HIV/AIDS Control, Ministry of Health, Hanoi, Vietnam; Medical University of Vienna, AUSTRIA

## Abstract

**Background:**

Methadone maintenance treatment (MMT) reduces HIV risk behaviors and improves access to HIV-related services among drug users. In this study, we assessed the uptake and willingness of MMT patients to refer HIV testing and counseling (HTC) service to their sexual partners and relatives.

**Methods:**

Health status, HIV-related risk behaviors, and HTC uptake and referrals of 1,016 MMT patients in Hanoi and Nam Dinh were investigated. Willingness to pay (WTP) for HTC was elicited using a contingent valuation technique. Interval and logistic regression models were employed to determine associated factors.

**Results:**

Most of the patients (94.2%) had received HTC, 6.6 times on average. The proportion of respondents willing to refer their partners, their relatives and to be voluntary peer educators was 45.7%, 35.3%, and 33.3%, respectively. Attending MMT integrated with HTC was a facilitative factor for HTC uptake, greater WTP, and volunteering as peer educators. Older age, higher education and income, and HIV positive status were positively related to willingness to refer partners or relatives, while having health problems (mobility, usual care, pain/discomfort) was associated with lower likelihood of referring others or being a volunteer. Over 90% patients were willing to pay an average of US $17.9 for HTC service.

**Conclusion:**

The results highlighted the potential role of MMT patients as referrers to HTC and voluntary peer educators. Integrating HIV testing with MMT services and applying users’ fee are potential strategies to mobilize resources and encourage HIV testing among MMT patients and their partners.

## Introduction

Expanding HIV testing among most-at-risk populations, including people who inject drug (PWID), female sex workers (FSW), men who have sex with men (MSM), and their sexual partners is critical to prevent HIV transmission and promotes early access to HIV-related care and treatment services in concentrated HIV epidemics [1]. However, there is still a high proportion of people who are at risk of HIV transmission are not aware their HIV status[2].

In 2014, the Joint United Nations Programme on HIV/AIDS (UNAIDS) declared the 90-90-90 targets for 2020, with the goal of identifying 90% PLWH living in community [3]. Regarding the UNAIDS target, HIV testing and counselling services (HTC) is a crucial component [4]. HTC can provide knowledge of current HIV status for clients, raise awareness of the importance to change HIV-related risk behaviors, and connecting positive individuals to HIV medical care if needed [5]. Empirical evidence has shown that HTC can reduce sexual risk behaviors among HIV positives [6] and eventually HIV incidence [7, 8]. Therefore, improving HTC uptake has an indispensable role in improving the efficiency and outcomes of HIV programs [9].

In Vietnam, scaling-up HTC services has been a priority in the National HIV/AIDS Strategic Plan [10, 11]. To date, there are 1,345 HTC clinics in Vietnam, providing services for 260,000 clients and about 227,000 HIV-positive cases have been reported [12]. However, many individuals still lack of awareness of their HIV status[13–15]. Results of Vietnam 2014 HIV/STI Sentinel Survey Plus Behavior indicated the low prevalence of HTC uptake in key populations, such as 38% in FSW and 39.4% in MSM [15]. Therefore, widespread introduction of HTC by diverse channels is necessary to improve the HTC accessibility [9].

As the country where HIV epidemic is largely driven by drug injection, the rapid expansion of methadone maintenance treatment (MMT) services over the past five years has brought about significant changes in HIV prevention and control [10, 12, 16–18]. Although methadone is known to reduce the frequency of drug use and inject[19–21], evidence for the reduction of unsafe sexual behaviors is equivocal[22–24]. Additionally, the low prevalence of HTC uptake among drug using population has been well documented (28%) [11, 15, 25, 26]. Therefore, sexual partners of drug users are at high risk of acquiring HIV. To address this issue, integrating HTC into MMT clinics and peer-delivered approaches has been hypothesized as a potentially effective approach [27, 28]. Literature indicates that PWID prefer HIV and Hepatitis C (HCV) testing services in methadone clinics rather than general or specialized health care clinics [29]. Furthermore, they are also willing to receive referral to HTC from their peers [27]. Thus, introducing MMT patients as referrers or peer educators may promote the use of HTC amongst their peers and sexual partners.

Currently, in Vietnam, voluntary HTC services are operated with 91% budget from international donors [30, 31]. Therefore, some HTC clinics offer free-of-charge services, while others require co-payment from clients with a price of VND 30,000–50,000 (US $1.5–2.5) without reimbursement by health insurance. This cost is much lower than the actual costs of HTCs. Prior literatures suggested that the mean cost for a HTC client in Vietnam is from US $7.6 to $30.3 [32, 33]. Since foreign aids for HIV programs in Vietnam are rapidly decreasing [34], transitioning the funding and management responsibility to the Vietnam Government is required in the next few years. It is estimated that the Government of Vietnam will need to spend US $32,269,698 for HTCs by 2020 [32]. Therefore, along with expanding its coverage, mobilizing resources from various sources, including copayment by service users, should be considered to ensure the sustainability of the HIV/AIDS programs.

The purposes of this study were to assess the HTC uptake and willingness of MMT patients to refer this service to and become peer educators for their sexual partners and relatives. In addition, patients’ willingness to pay for a HTC service was evaluated.

During the period of the study, voluntary HTC services were widely scaled up in the country with about 500 clinics[26]. Clients were provided HTC free-of-charge through supports of international donors. However, only a small proportion of high-risk populations had received HIV testing[35]. The study has been conducted during the period when international donors reduce their funding and transfer responsibility for financial support for HIV programs to the Vietnamese government. Co-payment for HIV services is therefore necessary to ensure sufficient resource for HIV interventions[16, 26]

## Methods

### Survey design and sampling procedure

From June to August, 2013 a cross-sectional study was conducted in Ha Noi and Nam Dinh province. There were five clinics involving in this study, including four facilities in district level (Tu Liem, Ha Dong, Long Bien, and Xuan Truong) and one clinic located at provincial level (Nam Dinh Provincial AIDS Center). The characteristics of study sites are listed in Table 1.

**Table 1:** Study settings and sample size.

Level	Settings	Site Name	Type of services	Sample size
Province	Nam Dinh City	Provincial AIDS Center	MMT+ HTC*	270
District (rural)	Xuan Truong District	District Health Center	MMT+ HTC + ART + GH*	151
District (urban)	Tu Liem District	District Health Center	MMT+ HTC + ART + GH*	201
District (urban)	Long Bien District	District Health Center	MMT+ HTC + ART + GH*	184
District (urban)	Ha Dong District	Regional Polyclinic	MMT+ GH*	210

* MMT: Methadone maintenance treatment; **HTC**: HIV testing and counseling service; **ART**: antiretroviral therapy; **GH:** General health care

In the study settings, some MMT clinics were co-located with HTC clinics but operated by separated management units (Table 1). Survey participants were comprised patients who were enrolled in MMT at selected sites. The eligibility criteria also included: 1) Age 18 years or older; 2) Visiting the clinics during the study period, and 3) Able to answer the interview questions. Patients were invited to a separate room to ensure privacy. If patients agreed to participate, they were asked to provide written inform consent. A convenient sample of 1,016 patients was enrolled in the study, accounting for 80–90% of the sample frame [36–39].

### Measures and instruments

Face-to-face interviews were conducted by well-trained interviewers who were MPH students. A structured questionnaire was used to collect data on socioeconomic characteristics, health status, drug use and sexual behaviors, HIV testing services utilization, and referrals.

#### Socio-economic information

Data about age, gender, occupation, education, religion and monthly income were self-reported. Monthly per capita household income was computed by summing all sources of income for each household member. Then this data was divided into five quintiles that were categorized from “poorest” to “richest”.

#### Health status

EuroQOL– 5 Dimensions– 5 levels (EQ-5D-5L) instrument was employed to measure health status of patients in five domains (mobility, self-care, usual activities, pain/discomfort and anxiety/depression) [40]. There were five levels of response in each domain from “No problem” to “Extremely problem”. Patients were classified into “Having problem” group if they reported “Slightly” to “Extremely”. This instrument has been widely used in Vietnam and proved to have good measurement properties in HIV-related populations [16, 41–45].

#### HIV-related risk behaviors

Risk behaviors of HIV transmission were collected regarding to drug use and sexual behaviors. The former comprised history of drug use and inject, drug treatment, drug use relapse, current drug use, and cost of drug use. The latter included information about number and type of sex partners, condom use, and percentage of condom use in the last 12 months. We also collected data about HIV status, ART use, and duration of MMT treatment.

#### HTC uptake, willingness to pay and referral

Outcomes of interest included the number of HTC events, patients’ willingness to pay (WTP) for a HTC service, and willingness to refer partners and relatives to HTC. To elicit patient’s WTP for HTC, a bidding game approach combining with open-ended question was used. First, interviewers summarized several aspects of HTC to ensure that patients had sufficient background knowledge before completing the willingness to pay valuation. Interviewers emphasized the benefits of testing for HIV when an individual perceived at-risk of HIV transmission as well as having pre- and post- test counseling. In addition, interviewers explained the importance of early access to antiretroviral services, including treatment of opportunistic infection, and referrals of individuals and their partners to HTC and HIV-related services.

Double-bounded dichotomous-choice questions backed by an open-ended question were used to elicit willingness to pay for HTC. This technique is used to reflect the actual behavior of individuals in regular markets [46]. In previous surveys, the cost per HTC visit ranged from US $38.9 in 2007 [33] to US $7.6 in 2012 [32] due to the fact that higher number of clients resulted in lower costs [32]. Therefore, to adapt those results and adjusted to the number of clients per site, an initial bid of 400 thousand VND (= US $20, 2013 rate) was applied.

Initially, each patient was first asked whether they were willing to pay 400 thousand VND (= US $20, 2013 rate) for HTC. If the patient was willing to pay US$ 20, the interviewer asked whether they were willing to pay double the initial price, or a half of the initial price. The question was repeated until the amount that the patient was willing to pay was four times or one fourth the initial price. Patients were then asked, “What is the maximum price you would be willing to pay for HTC?”

### Statistical analysis

Student *t* and *χ*^2^ tests were used to examine differences in characteristics of respondents. Because data on WTP was developed by the combination of censored and uncensored data, multivariate interval regression was employed to estimate the WTP for a HTC visit and its determinants. For HTC uptake and referral, we used multivariate logistic regression. Stepwise backward strategies were applied to construct the reduced model due to the log likelihood ratio test, with p-values > 0.2 for the threshold for exclusion.

### Ethical approval

Ethics approval of the study protocol was approved by the Vietnam Authority of HIV/AIDS Control's Scientific Research Committee. The data collection at study sites were approved and supported by Provincial AIDS Center in Ha Noi and Nam Dinh province. Written informed consent was obtained from all participants. Patients were informed that they could withdraw from the study at any time without influencing their current treatment.

## Results

The Table 2 shows the socio-economic status of 1,016 respondents. The age group 25–35 accounted for the majority of sample (52.4%). The predominance groups were those living with spouse (67.4%), attaining secondary school education (41.9%), being self-employed (53.4%), and ancestors worshiping (88.2%). Regarding health status, about 7.3%, 3.9%, and 5.9% had problems in mobility, self-care, and usual activities, respectively. The proportion of people having pain/discomfort and anxiety/depression were 17.7% and 20.7%, correspondingly.

**Table 2:** Demographics and health-related quality of life of respondents.

	Without HTC		With HTC		Total		p-value
	N	%	N	%	N	%	
**Age**							
18-<25	7	3.3	14	1.7	21	2.1	0.14
25-<30	33	15.7	106	13.2	139	13.7	
30- <35	53	25.2	213	26.4	266	26.2	
35- <40	43	20.5	223	27.7	266	26.2	
40- <45	42	20.0	125	15.5	167	16.4	
> = 45	32	15.2	125	15.5	157	15.5	
**Marital status**							
Single	47	22.4	204	25.3	251	24.7	0.66
Live with spouse	147	70.0	538	66.8	685	67.4	
Live with partner	1	0.5	2	0.3	3	0.3	
Divorced	15	7.1	57	7.1	72	7.1	
Widow	0	0.0	5	0.6	5	0.5	
**Educational attainment**							
Illiterate	4	1.9	13	1.6	17	1.7	0.93
Elementary	27	12.9	92	11.4	119	11.7	
Secondary	86	41.0	340	42.2	426	41.9	
High	81	38.6	306	38.0	387	38.1	
Vocational	7	3.3	25	3.1	32	3.2	
University	5	2.4	30	3.7	35	3.4	
**Employment**							
Unemployed	53	25.2	206	25.6	259	25.5	0.89
Self-employed	112	53.3	430	53.4	542	53.4	
White collars	5	2.4	17	2.1	22	2.2	
Workers, Farmers	18	8.6	82	10.2	100	9.8	
Students			2	0.3	2	0.2	
Other jobs	22	10.5	69	8.6	91	9.0	
**Religion**							
Cult of ancestors	198	94.3	698	86.6	896	88.2	<0.05
Buddhism	10	4.8	49	6.1	59	5.8	
Catholic	2	1.0	54	6.7	56	5.5	
Protestant	0	0.0	5	0.7	5	0.5	
**Health status**							
Having mobility problem	15	7.1	59	7.3	74	7.3	0.93
Having self-care problem	9	4.3	31	3.9	40	3.9	0.77
Having usual activities problem	9	4.3	51	6.3	60	5.9	0.26
Having pain/discomfort	34	16.2	146	18.1	180	17.7	0.52
Having anxiety/depression	38	18.1	172	21.3	210	20.7	0.30

As presented in Table 3, most of the sample (98.8%) had sexual intercourse at least once in the prior year, and the majority of respondents had one sexual partner (69.7%). The main type of sex partner was primary partners (spouse or boy/girlfriend) (78.7%); while a small percentage of patients had sexual contact with casual sexual partners (6.0%) or commercial sex workers (8.1%). The percentage of people having sexual intercourse with primary partners, casual partners, and sex workers without condoms was 71.9%, 42.6%, and 15.9%, respectively. In addition, the mean percentage of condom use with primary partners among MMT patients was the lowest with 24.2% (SD = 39.3%) compared to with casual partners or sex workers.

**Table 3:** Sexual behaviors among respondents.

	Without HTC		With HTC		Total		p-value
	N	%	N	%	N	%	
**Ever had sex**	210	100.0	794	98.5	1,004	98.8	0.07
**Number of sexual partners (in the last 12 months)**							
Not had anyone	24	11.4	159	19.7	183	18.0	<0.05
One sex partners	156	74.3	552	68.5	708	69.7	
2–3 sex partners	13	6.2	33	4.1	46	4.5	
>4 sex partners	17	8.1	62	7.7	79	7.8	
**Type of sex partner**							
Primary partners	172	81.9	628	77.9	800	78.7	0.21
Casual sex partners	13	6.2	48	6.0	61	6.0	0.90
Sex workers	12	5.7	70	8.7	82	8.1	0.16
**Inconsistent condom use**							
With Primary sexual partners (n = 800)	142	82.6	433	69.0	575	71.9	<0.001
With Casual sexual partners (n = 61)	9	69.2	17	35.4	26	42.6	<0.05
With Sex workers (n = 82)	1	8.3	12	17.1	13	15.9	0.44
**Percentage of condom use (in the last 12 months)**	**Mean**	**SD**	**Mean**	**SD**	**Mean**	**SD**	
With Primary sex partners	16.0	33.1	26.5	40.5	24.2	39.3	<0.001
With Casual sexual partners	20.8	40.1	29.2	45.9	27.4	44.6	0.28
With Sex workers	33.3	49.2	30.7	45.9	86.4	33.2	0.43

Table 4 illustrated drug use behaviors among MMT patients. Only 4.8% currently reported use of illicit drug. About three out of four respondents had drug injecting experience with the mean age of initial injection of age 26.8 (95%CI = 26.3–27.4). Most of them had drug detoxification treatment at least one time (92.7%) and the major location for rehabilitation was at home (70.1%). The primary reasons for relapse were peer influence (47.7%) and craving (43.2%). The results indicate that 8.1% were HIV positive and 6.5% were on ART. The mean duration of MMT treatment was 16.6 (95% 15.9–17.3) months.

**Table 4:** Drug use behaviors among respondents.

Characteristics	Without HTC			With HTC			Total			p-value
	N	%		N	%		N	%		
**Ever inject drug**	161	76.7		585	72.6		746	73.4		0.23
**Current drug use**	11	5.2		38	4.7		49	4.8		0.75
**# drug rehabilitation**										
None	18	8.6		56	7.0		74	7.3		0.09
1–5 episodes	151	71.9		524	65.0		675	66.4		
6–10	33	15.7		178	22.1		211	20.8		
>10	8	3.8		48	6.0		56	5.5		
**Location of previous drug rehabilitation**										
Home	141	67.1		545	70.9		686	70.1		0.30
Private voluntary center	99	47.1		354	46.2		453	46.4		0.80
Compulsory center	33	15.7		224	29.2		257	26.3		<0.001
**Reason for relapsed**										
Boredom	81	38.6		285	35.4		366	36.0		0.39
Peer inducement	113	53.8		372	46.2		485	47.7		0.05
Craving	99	47.1		340	42.2		439	43.2		0.20
Unemployment	6	2.9		43	5.3		49	4.8		0.14
**HIV positive**	9	4.3		73	9.1		82	8.1		<0.05
**ART**	6	2.9		60	7.4		66	6.5		<0.05
	**Mean**	**95% CI**		**Mean**	**95% CI**		**Mean**	**95% CI**		
**# previous drug rehabilitation episodes**	4.6	3.9	5.4	5.5	5.0	6.1	5.3	4.9	5.8	<0.05
**Age at first drug use**	25.3	24.4	26.2	24.3	23.9	24.8	24.5	24.1	25.0	<0.05
**Age at first drug injection**	27.9	26.8	29.0	26.6	26.0	27.2	26.8	26.3	27.4	<0.05
**Time since 1st drug use (years)**	12.2	11.4	13.0	13.6	13.2	14.0	13.3	12.9	13.6	<0.05
**Time since first drug injection (years)**	9.2	8.5	9.9	10.5	10.1	10.9	10.2	9.9	10.6	<0.05
**Daily cost of drug use (1000 vnd)**	356.4	244.7	468.1	322.3	277.5	367.1	326.8	285.4	368.2	0.29
**Duration on MMT (month)**	15.2	14.1	16.3	16.9	16.1	17.8	16.6	15.9	17.3	<0.001

HTC uptake, referrals, and willingness to pay are shown in Table 5. Of the sample, 94.2% had ever used HTC, and the mean number of HIV tests was 6.6 (95%CI = 5.6–7.6). Health workers was the primary source of referrals for the first HTC (59.6%). The findings show that 45.7% and 35.3% of respondents were willing to refer partners and other relatives to HIV testing, respectively. Furthermore, 33.3% patients would volunteer to be peer educators. The proportion of people being willing to pay for HTC was 91.6%, and the amount of WTP was 358 thousand VND per visit (95%CI = 332–385 thousand). The amount of WTP among people in clinics having HTC was significantly higher than their counterparts (p<0.05).

**Table 5:** HTC uptake and willingness to pay among respondents.

Characteristics	Without HTC			With HTC			Total			p-value
	n	%		n	%		n	%		
**Ever use HTC**	206	98.1		751	93.2		957	94.2		<0.05
**Referrer of the first HTC used**										
Spouse	4	1.9		24	3.2		28	2.9		<0.05
Peers	5	2.4		33	4.4		38	4.0		
Health workers	149	72.3		421	56.1		570	59.6		
Media	1	0.5		12	1.6		13	1.4		
Self-motivation	42	20.4		226	30.1		268	28.0		
Parents/Relatives	5	2.4		35	4.7		40	4.2		
**HIV status of relatives (Positive)**										
Family members	6	2.9		40	5.0		46	4.5		0.19
Spouse/Partners	2	1.0		3	0.4		5	0.5		0.28
Parents	0	0.0		1	0.1		1	0.1		0.61
Brother/Sister	2	1.0		4	0.5		6	0.6		0.44
Kinship	0	0.0		5	0.6		5	0.5		0.25
Other relatives	1	0.5		0	0.0		1	0.1		0.05
**Refer partners to HIV testing services**	85	40.5		379	47.0		464	45.7		0.1
**Refer other relatives to HIV testing services**	70	33.3		289	35.9		359	35.3		0.5
**Volunteer to be a Peer educator**	52	24.8		286	35.5		338	33.3		<0.05
**Willing to pay for HTC**	188	89.5		743	92.2		931	91.6		0.22
	**Mean**	**95% CI**		**Mean**	**95% CI**		**Mean**	**95% CI**		
**# HTC test uptake**	4.3	0.7	8.0	6.8	5.8	7.9	6.6	5.6	7.6	0.09
**Willingness to pay for a HTC (thousand VND)**	304	252	355	373	343	403	358	332	385	0.04

Table 6 shows the reduced models of the multivariate interval and logistic regression. Participants were willing to pay more for a HTC visit if they were 40–45 years old; had higher levels of education, higher monthly income, and volunteered to be a peer educator. Having usual activities problem and pain/discomfort were associated with willing to pay less than others. The data in Table 6 also demonstrates a negative relation between the number of HIV test uptake and living with spouse, while the positive associations were linked to being widowed, employment, higher income, HIV positive status, using MMT service without HTC, being self-referred to the first HTC use and referring partners to HTC.

**Table 6:** Factors associated with the use of and WTP for HTCs and referrals for sexual partners and other relatives among MMT patients.

Characteristics	Willingness to pay for HTC			# HIV test uptake			Refer partners to HTD			Refer other relatives to HTC			Volunteer to be a Peer educator		
	Coef	95% CI		Coef	95% CI		OR	95% CI		OR	95% CI		OR	95% CI	
**Sex (Female vs. Male)**										2.4	0.7	8.5			
**Age (**18- <25—ref)															
25- <30													0.3*	0.1	0.8
30- <35										0.8	0.5	1.0	0.3*	0.1	0.9
35- <40				0.3	-0.1	0.7							0.3*	0.1	0.8
40- <45	82.6*	10.8	154.3										0.4	0.1	1.2
> = 45										0.7	0.5	1.1	0.2*	0.1	0.6
**Marital status** (Single-ref)															
Living with spouse				-0.5*	-1.0	0.0	1.6*	1.1	2.3						
Divorced															
Widow				2.9*	0.2	5.6	1.8	1.0	3.3	9.7	0.9	100.6			
**Religion** (Cult of ancestors–ref)							11.5	1.0	130.0						
Catholic				-0.8	-1.6	0.1									
Protestant													5.8	0.6	52.8
**Education** (Illiterate–ref)															
Elementary	103.9*	15.0	192.8							0.6	0.4	1.0	0.6*	0.4	1.0
Secondary	84.9*	27.7	142.0				1.5*	1.0	2.2						
Vocational	210.2*	55.4	365.0				1.5*	1.0	2.3						
University				-0.7	-1.8	0.4									
**Employment** (Unemployed–ref)															
Self-employed							1.4	1.0	1.8	1.3	0.9	1.7			
White collars	127.1	-51.3	305.5				2.9*	1.1	7.9	2.8*	1.1	7.0	1.8	0.7	4.5
Other jobs				0.8*	0.1	1.5				0.7	0.4	1.2			
**Income per capita** (Poorest–ref)															
Poor				0.4	-0.2	0.9	0.8	0.5	1.1						
Middle	80.9*	12.5	149.4	0.4	-0.2	0.9	0.7	0.5	1.0						
Rich	98.3*	31.3	165.3	0.7*	0.2	1.3									
**HIV status (Positive vs. Negative)**				1.2*	0.5	1.9	2.3*	1.3	4.0	2.9*	1.7	4.9			
**Kinship HIV status (Positive vs. Negative)**										5.2	0.7	38.0			
**Mobility (Have problems vs. No problems)**													0.4*	0.2	0.7
**Self-care (Have problems vs. No problems)**										2.0	0.8	4.7			
**Usual Activities (Have problems vs. No)**	-118.8*	-234.2	-3.5							0.5	0.2	1.1	2.8	1.4	5.8
**Pain/Discomfort (Have problems vs. No)**	-108.1*	-181.4	-34.7				0.7	0.5	1.0	0.6*	0.4	0.9	0.7	0.4	1.0
**MMT service model (with HTC vs. without HTC)**	45.3	-19.6	110.2	0.6*	0.1	1.1							1.9*	1.3	2.8
**Referrer of the first HTC used** (Spouse-ref)															
Peers				0.9	-0.1	1.8							2.7*	1.4	5.5
Health workers	-105.2*	-187.6	-22.7				0.5*	0.3	0.8						
Media				1.2	-0.4	2.9							2.3	0.7	7.2
Self-motivation	-60.2	-149.5	29.1	0.4*	0.0	0.9	0.7	0.4	1.1	1.3	0.9	1.8			
**Current drug use (Yes vs. No)**	86.1	-41.8	214.1				0.5	0.3	1.1						
**Condom use with primary partner (**Yes-ref)															
No							0.7	0.5	1.0	0.8	0.5	1.1			
Not have sexual intercourse				-0.5	-1.1	0.1	0.4	0.2	0.6	0.5*	0.4	0.8			
**Condom use with Casual sexual partners (**Yes-ref)															
No										5.5	0.4	69.2			
Not have sexual intercourse										1.7	0.9	3.2			
**Times taking HTC**							1.1*	1.1	1.2	1.1*	1.0	1.1			
**Refer partners to HTC (Yes vs. No)**				1.0*	0.6	1.4									
**Refer other relatives to HTC (Yes vs. No)**	-57.8	-115.9	0.2												
**Volunteer to be a Peer educator (Yes vs. No)**	128.8*	68.9	188.8	-0.4	-0.8	0.0									
**Constant**	297.6	188.4	406.9	2.3	1.6	3.0	0.7	0.4	1.4	0.3	0.2	0.7	1.1	0.4	3.2

* p<0,05

Respondents were more likely to be willing to refer partners to HTCs if they were they had white collar occupations, lived with a spouse, and had a higher level of education. In addition, the similar tendencies were observed among people living with HIV and those who had more frequently used HTC. In contrast, patients who were referred to the first HTC used by health workers were less likely to be willing to refer partners. In regards to willingness to refer other relatives to HTC, having a white collar occupation, HIV positive status, and higher number of HTC experiences were facilitating factors; while having pain/discomfort and not having sexual intercourse with primary partners (spouse/beloved) were inversely associated with willingness to refer of other relatives.

Table 6 indicates that respondents who were older, had an elementary education, and mobility problems were less likely to volunteer to be peer educators and people in MMT service without HTC or being referred to the first HTC use by peers were more likely to volunteer.

## Discussion

In our knowledge, this is the first study investigating the role of MMT patients on HTC referral and resource mobilization in Vietnam. The findings may inform policy development to scale-up the coverage of HTC amongst drug users, their sexual partners, and peers. We a high level of WTP for HTCs among MMT patient. Furthermore, almost half of respondents were willing to refer to their partners/relatives and more than one third of them were willing to be voluntary peer educators. Adjusting to other factors, providing HTC integrated with MMT sites appeared to facilitate HTC uptake and interest in referring to peers to HTC among drug using populations.

### HTC uptake

Most of the respondents (94.2%) reported ever receiving HTCs. These result were around much higher than the rate of HTC uptake in general drug use population (28.0%) and other high risks populations such as female sex workers (38%) or men who have sex with men (39.4%) [15]. This findings can be explained by the fact that MMT patients in Vietnam were selective as the availability of services was still limited, and those who were in MMT may have had strong motivation and supports from their families. Our result was also higher than HTC uptake of MMT patients in China (75.7%) [28], Indonesia (44%) [47] and USA (34%) [48]. MMT has been shown to reduce frequency of HIV risk behavior and increase the use of HIV-related services [18, 21, 49]. This study contributes to the literature by demonstrating that enrollment in MMT may empower patients to be catalysts for accelerating the expansion of HIV testing amongst at risk populations. In addition, previous research indicated that HIV testing results did not influence MMT retention but provided information for drug users to avoid transmitting HIV and early access to HIV care and treatment services [50]. Therefore, increasing the coverage of MMT program may have a significant role in expanding the coverage of HTC.

Patients participating in an integrative MMT-HTC clinic were found to have higher number of HTC visits compared to others. In some settings, providing HTC at MMT clinics may eliminate several barriers for uptake such as distance and lack of transports [51, 52]. Easy to access HTC promotes uptake among drug users and routine HIV testing is recommended in MMT clinics [4, 48]. However, protecting confidentiality in integrative HTC models should be addressed. Some studies illustrated that in this model, confidentiality might be at risk due to the lack of privacy, staff training, and power differentials between providers and clients [53–56], while other surveys report opposite results [57, 58].

### HTC referrals

Since PWID sexual partners are at high risk of HIV infection [59, 60], present study indicates the feasibility of MMT patients referring their sexual partners to HTC. A review of Hogben revealed that partner referral was an effective way to identify HIV-positive case[61]. However, in Vietnam, a study of Hong et al. showed that only 1.9% clients were referred by sexual partners and only one of four clients utilized HTC because their sexual partners were HIV-infected or in high-risk populations [62]. In our study, almost half of respondents were willing to refer partners/relatives to HTC and one third of sample was also willing to be voluntary peer educators. Those findings suggested a potential referral channel for promoting HTC among approach hidden populations.

Some facilitative factors for HTC referrals in this study were living with spouse, higher education, having white collar jobs, HIV-positive status, and greater number of HTC experiences. Indeed, those facilitators were frequently related with high level of knowledge and attitudes about HIV and HTC [63–65]; hence, those with greater knowledge may perceived the importance of HTC and were more willing to refer their partners/relatives. Moreover, people who used the integrative clinic model were more likely to express willingness to be voluntary peer educators. As demonstrated, integrative MMT-HTC clinics could improve HTC utilization. It may help patients to understand clearly the important role of HTC to prevent HIV transmission among drug use population, and then, encourage drug users to be voluntary peer educators.

Additionally, the results suggest that patients receiving referrals from peers for the first HTC use were more willing to be voluntary peer instructors. In HIV program, peer education is regularly used to prevent HIV infection and other sexual transmitted infections [66]. Peer educators had similar characteristics or behaviors with high risk population, therefore they may have high level of trust and comfort with their peers [67]. Moreover, they can more easily access hidden HIV population as compared to other approaches [68]. Thus, the role of peer educators is critical in encouraging HTC uptake and referrals of MMT patients.

Notably, having pain/discomfort was recognized as a barrier preventing the referral to relatives among MMT patients. Likewise, having mobility problem was negatively related with being voluntary peer instructors. The reason for this association is not be clear, but a study of Yang et al. from China showed that people having poor health status had more negative attitudes toward HTC than those having better health status [69], thus they did not want to refer and to volunteer. Collectively, addressing health problems of MMT patients should be considered to promote HTC referrals among this population.

### Willingness to pay for HTC

In this study, we observed an enormous proportion of patients willing to pay for HTC (91.6%) with the mean amount of US $17.9. Although this amount of WTP seems to be higher than the current user fees applied for HTC in Vietnam, it was about a half of the economic cost for the service in 2007 (US $38.9) [33]. This high level of willingness can be explained by the fact that our sample was primarily HIV-negative drug users who valued HTC and had strong motivation and familial supports to change their health behaviors. Consistent with previous studies, factors associated with WTP for HTC included older age and higher education and income [16, 70–74]. Furthermore, those who were willing to volunteer to be peer educator were also willing to pay more. In prior literatures, positive attitudes for a product demonstrates a strong association with WTP [75–77]. Conversely, people having usual-activities and pain/discomfort problems were observed to have lower amounts of WTP for HTC, which was consistent with previous studies that poor health status could be a negative factor of WTP [16, 78].

### Implications

This study had several implications. First, HTC should be integrated with MMT clinics to encourage testing and referrals among drug users and their sexual partners and peers. This integrative model could also help to reduce the duplicated operation cost by provider and transportation costs by users. Second, providing medical care promptly to patients having health problems is likely to improve their interest in providing referrals or being voluntary peer educators and increase willingness to pay for HTC. Third, peer educators in clinics are needed to promote patients’ HTC. Voluntary peer instructors may be effective by sharing experience and encouraging participating in peer educator groups. Those factors mentioned above could help to effectively expand HTC to other at risk populations. Future studies may be useful to assess if relatives and partners of MMT patients have greater awareness on HIV/AIDS, maintain healthy behaviors, and engage in HTC. Finally, the result suggests that resources mobilization through users’ fees for VCT may be applied to ensure the sustainability of HIV/AIDS program. During the study period, co-payment for HTC has been piloted in some HTC clinics in Vietnam with US $1.5 to 2.5 per visit. The amount that patients were willing to pay for HTC in this paper was hypothetical. Further translational research is needed on actual WTP before becoming the basis for HTC policy. Also, it is important to note that the application of user fee for HIV testing could serve as a barrier for the most vulnerable and highest risk segment of the MMT population. Our findings suggest that the justification of user fee can be based on economic cost analyses with continuous subsidy for socioeconomically disadvantaged groups. Additionally, due to the fact that the cost per HTC uptake is depend on the number of clients, program managers should use performance-based incentives for peer instructors to refer targeted populations to HTC [32].

### Limitations

This study has several limitations. First, recall bias may be occurred in self-reported data. Second, the sensitive questions about drug use or sexual behaviors be subject to social desirability bias. Furthermore, cross-sectional study may limit the establishment of causal relations between HTC uptake, referral, willingness to pay and MMT treatment. Finally, the convenient sample may limit generalizability of the findings to a larger population.

## Conclusions

In conclusion, this study showed a high willingness of MMT patients to pay for HTC. Moreover, about a half of the patients were willing to refer their partners and relatives to HTC services, and one third to be voluntary peer instructors. Integrating HIV testing with MMT services and applying user’s fee are potential strategies to mobilize resources and encourage HIV testing among MMT patients and their partners.

## Supporting Information

S1 File Supporting Information MMT HIV testing referrals (6) 31012016.doc. (DOC)
