# Supplementary material for: Methadone Maintenance Treatment Promotes Referral and Uptake of HIV Testing and Counselling Services amongst Drug Users and Their Partners
Source: PLoS One. 2016 Apr 5;11(4):e0152804. doi: 10.1371/journal.pone.0152804 (PMC4821610; doi:10.1371/journal.pone.0152804)
Supplement: S1 File — (DOC) [file pone.0152804.s001.doc]

**Methadone maintenance treatment promotes referral and uptake of HIV testing and counselling services amongst drug users and their partners**

Bach Xuan Tran^1,2^, Long Hoang Nguyen^1,3^, Lan Phuong Nguyen^4^, Cuong Tat Nguyen^5^, Huong Thu Thi Phan^6^, Carl A. Latkin^2^

^1^Institute for Preventive Medicine and Public Health, Hanoi Medical University, Hanoi, Vietnam

^2^Johns Hopkins Bloomberg School of Public Health, Baltimore, Maryland, United States of America

^3^School of Medicine and Pharmacy, Vietnam National University, Hanoi, Vietnam

^4^Harvard T.H Chan School of Public Health, United States of America

^5^Institute for Global Health Innovations, Duy Tan University, Da Nang, Vietnam

^6^Authority of HIV/AIDS Control, Ministry of Health, Hanoi, Vietnam

Bach Xuan Tran and Long Hoang Nguyenequally contributed.

Corresponding author:

Bach Tran, PhD

Lecturer in Health Economics
Hanoi Medical University, Vietnam

Assistant Professor (Adjunct)

Bloomberg School of Public Health

Johns Hopkins University, USA

**Abstract**

**Background:** Methadone maintenance treatment (MMT) reduces HIV risk behaviors and improves access to HIV-related services among drug users. In this study, we assessed the uptake and willingness of MMT patients to refer HIV testing and counseling (HTC) service to their sexual partners and relatives.

**Methods:** Health status, HIV-related risk behaviors, and HTC uptake and referrals of 1,016 MMT patients in Hanoi and Nam Dinh were investigated. Willingness to pay (WTP) for HTC was elicited using a contingent valuation technique. Interval and logistic regression models were employed to determine associated factors.

**Results:** Most of the patients (94.2%) had received HTC, 6.6 times on average. The proportion of respondents willing to refer their partners, their relatives and to be voluntary peer educators was 45.7%, 35.3%, and 33.3%, respectively. Attending MMT integrated with HTC was a facilitative factor for HTC uptake, greater WTP, and volunteering as peer educators. Older age, higher education and income, and HIV positive status were positively related to willingness to refer partners or relatives, while having health problems (mobility, usual care, pain/discomfort) was associated with lower likelihood of refer othering or being a volunteer. Over 90% patients were willing to pay an average of US $17.9 for HTC service.

**Conclusion:** The results highlighted the potential role of MMT patients as referrers to HTC and voluntary peer educators. Integrating HIV testing with MMT services and applying users’ fee are potential strategies to mobilize resources and encourage HIV testing among MMT patients and their partners.

**Keywords:** HIV/AIDS, testing, counselling, referral, methadone maintenance.

**INTRODUCTION**

Expanding HIV testing among most-at-risk populations, including people who inject drug (PWID), female sex workers (FSW), men who have sex with men (MSM), and their sexual partners is critical to prevent HIV transmission and promotes early access to HIV-related care and treatment services in concentrated HIV epidemics [^1^](#_ENREF_1). However, there is still a high proportion of people who are at risk of HIV transmission are not aware their HIV status[^2^](#_ENREF_2).

In 2014, the Joint United Nations Programme on HIV/AIDS (UNAIDS) declared the 90-90-90 targets for 2020, with the goal of identifying 90% PLWH living in community [^3^](#_ENREF_3). Regarding the UNAIDS target, HIV testing and counselling services (HTC) is a crucial component [^4^](#_ENREF_4). HTC can provide knowledge of current HIV status for clients, raise awareness of the importance to change HIV-related risk behaviors, and connecting positive individuals to HIV medical care if needed [^5^](#_ENREF_5). Empirical evidence has shown that HTC can reduce sexual risk behaviors among HIV positives [^6^](#_ENREF_6) and eventually HIV incidence . Therefore, improving HTC uptake has an indispensable role in improving the efficiency and outcomes of HIV programs [^9^](#_ENREF_9).

In Vietnam, scaling-up HTC services has been a priority in the National HIV/AIDS Strategic Plan . To date, there are 1,345 HTC clinics in Vietnam, providing services for 260,000 clients and about 227,000 HIV-positive cases have been reported [^12^](#_ENREF_12). However, many individuals still lack of awareness of their HIV status[^13-15^](#_ENREF_13). Results of Vietnam 2014 HIV/STI Sentinel Survey Plus Behavior indicated the low prevalence of HTC uptake in key populations, such as 38% in FSW and 39.4% in MSM [^15^](#_ENREF_15). Therefore, widespread introduction of HTC by diverse channels is necessary to improve the HTC accessibility [^9^](#_ENREF_9).

As the country where HIV epidemic is largely driven by drug injection, the rapid expansion of methadone maintenance treatment (MMT) services over the past five years has brought about significant changes in HIV prevention and control . Although methadone is known to reduce the frequency of drug use and inject[^19-21^](#_ENREF_19), evidence for the reduction of unsafe sexual behaviors is equivocal[^22-24^](#_ENREF_22). Additionally, the low prevalence of HTC uptake among drug using population has been well documented (28%) . Therefore, sexual partners of drug users are at high risk of acquiring HIV. To address this issue, integrating HTC into MMT clinics and peer-delivered approaches has been hypothesized as a potentially effective approach . Literature indicates that PWID prefer HIV and Hepatitis C (HCV) testing services in methadone clinics rather than general or specialized health care clinics [^29^](#_ENREF_29). Furthermore, they are also willing to receive referral to HTC from their peers [^27^](#_ENREF_27). Thus, introducing MMT patients as referrers or peer educators may promote the use of HTC amongst their peers and sexual partners.

Currently, in Vietnam, voluntary HTC services are operated with 91% budget from international donors . Therefore, some HTC clinics offer free-of-charge services, while others require co-payment from clients with a price of VND 30,000-50,000 (US $1.5-2.5) without reimbursement by health insurance. This cost is much lower than the actual costs of HTCs. Prior literatures suggested that the mean cost for a HTC client in Vietnam is from US $7.6 to $30.3 . Since foreign aids for HIV programs in Vietnam are rapidly decreasing [^34^](#_ENREF_34), transitioning the funding and management responsibility to the Vietnam Government is required in the next few years. It is estimated that the Government of Vietnam will need to spend US $32,269,698 for HTCs by 2020 [^32^](#_ENREF_32). Therefore, along with expanding its coverage, mobilizing resources from various sources, including copayment by service users, should be considered to ensure the sustainability of the HIV/AIDS programs.

The purposes of this study were to assess the HTC uptake and willingness of MMT patients to refer this service to and become peer educators for their sexual partners and relatives. In addition, patients’ willingness to pay for a HTC service was evaluated.

During the period of the study, voluntary HTC services were widely scaled up in the country with about 500 clinics[^26^](#_ENREF_26). Clients were provided HTC free-of-charge through supports of international donors. However, only a small proportion of high-risk populations had received HIV testing[^35^](#_ENREF_35). The study has been conducted during the period when international donors reduce their funding and transfer responsibility for financial support for HIV programs to the Vietnamese government. Co-payment for HIV services is therefore necessary to ensure sufficient resource for HIV interventions

**METHODS**

Survey design and sampling procedure

From June to August, 2013 a cross-sectional study was conducted in Ha Noi and Nam Dinh province. There were five clinics involving in this study, including four facilities in district level (Tu Liem, Ha Dong, Long Bien, and Xuan Truong) and one clinic located at provincial level (Nam Dinh Provincial AIDS Center). The characteristics of study sites are listed in Table 1.

In the study settings, some MMT clinics were co-located with HTC clinics but operated by separated management units (Table 1). Survey participants were comprised patients who were enrolled in MMT at selected sites. The eligibility criteria also included: 1) Age 18 years or older; 2) Visiting the clinics during the study period, and 3) Able to answer the interview questions. Patients were invited to a separate room to ensure privacy. If patients agreed to participate, they were asked to provide written inform consent. A convenient sample of 1,016 patients was enrolled in the study, accounting for 80-90% of the sample frame [^36-39^](#_ENREF_36).

Measures and instruments

Face-to-face interviews were conducted by well-trained interviewers who were MPH students. A structured questionnaire was used to collect data on socioeconomic characteristics, health status, drug use and sexual behaviors, HIV testing services utilization, and referrals.

***Socio-economic information***

Data about age, gender, occupation, education, religion and monthly income were self-reported. Monthly per capita household income was computed by summing all sources of income for each household member. Then this data was divided into five quintiles that were categorized from “poorest” to “richest”.

***Health status***

EuroQOL – 5 Dimensions – 5 levels (EQ-5D-5L) instrument was employed to measure health status of patients in five domains (mobility, self-care, usual activities, pain/discomfort and anxiety/depression) [^40^](#_ENREF_40). There were five levels of response in each domain from “No problem” to “Extremely problem”. Patients were classified into “Having problem” group if they reported “Slightly” to “Extremely”. This instrument has been widely used in Vietnam and proved to have good measurement properties in HIV-related populations .

***HIV-related risk behaviors***

Risk behaviors of HIV transmission were collected regarding to drug use and sexual behaviors. The former comprised history of drug use and inject, drug treatment, drug use relapse, current drug use, and cost of drug use. The latter included information about number and type of sex partners, condom use, and percentage of condom use in the last 12 months. We also collected data about HIV status, ART use, and duration of MMT treatment.

***HTC uptake, willingness to pay and referral***

Outcomes of interest included the number of HTC events, patients’ willingness to pay (WTP) for a HTC service, and willingness to refer partners and relatives to HTC. To elicit patient’s WTP for HTC, a bidding game approach combining with open-ended question was used. First, interviewers summarized several aspects of HTC to ensure that patients had sufficient background knowledge before completing the willingness to pay valuation. Interviewers emphasized the benefits of testing for HIV when an individual perceived at-risk of HIV transmission as well as having pre- and post- test counseling. In addition, interviewers explained the importance of early access to antiretroviral services, including treatment of opportunistic infection, and referrals of individuals and their partners to HTC and HIV-related services.

Double-bounded dichotomous-choice questions backed by an open-ended question were used to elicit willingness to pay for HTC. This technique is used to reflect the actual behavior of individuals in regular markets [^46^](#_ENREF_46). In previous surveys, the cost per HTC visit ranged from US $38.9 in 2007 [^33^](#_ENREF_33) to US $7.6 in 2012 [^32^](#_ENREF_32) due to the fact that higher number of clients resulted in lower costs [^32^](#_ENREF_32). Therefore, to adapt those results and adjusted to the number of clients per site, an initial bid of 400 thousand VND (= US $20, 2013 rate) was applied.

Initially, each patient was first asked whether they were willing to pay 400 thousand VND (= US $20, 2013 rate) for HTC. If the patient was willing to pay US$ 20, the interviewer asked whether they were willing to pay double the initial price, or a half of the initial price. The question was repeated until the amount that the patient was willing to pay was four times or one fourth the initial price. Patients were then asked, “What is the maximum price you would be willing to pay for HTC?”

Statistical analysis

Student *t* and *χ*^2^ tests were used to examine differences in characteristics of respondents. Because data on WTP was developed by the combination of censored and uncensored data, multivariate interval regression was employed to estimate the WTP for a HTC visit and its determinants. For HTC uptake and referral, we used multivariate logistic regression. Stepwise backward strategies were applied to construct the reduced model due to the log likelihood ratio test, with p-values > 0.2 for the threshold for exclusion.

**Ethical approval**

Ethics approval of the study protocol was approved by the Vietnam Authority of HIV/AIDS Control's Scientific Research Committee. The data collection at study sites were approved and supported by Provincial AIDS Center in Ha Noi and Nam Dinh province. Written informed consent was obtained from all participants. Patients were informed that they could withdraw from the study at any time without influencing their current treatment.

**RESULTS**

The table 2 shows the socio-economic status of 1,016 respondents. The age group 25-35 accounted for the majority of sample (52.4%). The predominance groups were those living with spouse (67.4%), attaining secondary school education (41.9%), being self-employed (53.4%), and ancestors worshiping (88.2%). Regarding health status, about 7.3%, 3.9%, and 5.9% had problems in mobility, self-care, and usual activities, respectively. The proportion of people having pain/discomfort and anxiety/depression were 17.7% and 20.7%, correspondingly.

As presented in table 3, most of the sample (98.8%) had sexual intercourse at least once in the prior year, and the majority of respondents had one sexual partner (69.7%). The main type of sex partner was primary partners (spouse or boy/girlfriend) (78.7%); while a small percentage of patients had sexual contact with casual sexual partners (6.0%) or commercial sex workers (8.1%). The percentage of people having sexual intercourse with primary partners, casual partners, and sex workers without condoms was 71.9%, 42.6%, and 15.9%, respectively. In addition, the mean percentage of condom use with primary partners among MMT patients was the lowest with 24.2% (SD=39.3%) compared to with casual partners or sex workers.

Table 4 illustrated drug use behaviors among MMT patients. Only 4.8% currently reported use of illicit drug. About three out of four respondents had drug injecting experience with the mean age of initial injection of age 26.8 (95%CI=26.3-27.4). Most of them had drug detoxification treatment at least one time (92.7%) and the major location for rehabilitation was at home (70.1%). The primary reasons for relapse were peer influence (47.7%) and craving (43.2%). The results indicate that 8.1% were HIV positive and 6.5% were on ART. The mean duration of MMT treatment was 16.6 (95% 15.9-17.3) months.

HTC uptake, referrals, and willingness to pay are shown in Table 5. Of the sample, 94.2% had ever used HTC, and the mean number of HIV tests was 6.6 (95%CI=5.6-7.6). Health workers was the primary source of referrals for the first HTC (59.6%). The findings show that 45.7% and 35.3% of respondents were willing to refer partners and other relatives to HIV testing, respectively. Furthermore, 33.3% patients would volunteer to be peer educators. The proportion of people being willing to pay for HTC was 91.6%, and the amount of WTP was 358 thousand VND per visit (95%CI = 332-385 thousand). The amount of WTP among people in clinics having HTC was significantly higher than their counterparts (p<0.05).

Table 6 shows the reduced models of the multivariate interval and logistic regression. Participants were willing to pay more for a HTC visit if they were 40-45 years old; had higher levels of education, higher monthly income, and volunteered to be a peer educator. Having usual activities problem and pain/discomfort were associated with willing to pay less than others. The data in Table 6 also demonstrates a negative relation between the number of HIV test uptake and living with spouse, while the positive associations were linked to being widowed, employment, higher income, HIV positive status, using MMT service without HTC, being self-referred to the first HTC use and referring partners to HTC.

Respondents were more likely to be willing to refer partners to HTCs if they were they had white collar occupations, lived with a spouse, and had a higher level of education. In addition, the similar tendencies were observed among people living with HIV and those who had more frequently used HTC. In contrast, patients who were referred to the first HTC used by health workers were less likely to be willing to refer partners. In regards to willingness to refer other relatives to HTC, having a white collar occupation, HIV positive status, and higher number of HTC experiences were facilitating factors; while having pain/discomfort and not having sexual intercourse with primary partners (spouse/beloved) were inversely associated with willingness to refer of other relatives.

Table 6 indicates that respondents who were older, had an elementary education, and mobility problems were less likely to volunteer to be peer educators and people in MMT service without HTC or being referred to the first HTC use by peers were more likely to volunteer.

**DISCUSSION**

In our knowledge, this is the first study investigating the role of MMT patients on HTC referral and resource mobilization in Vietnam. The findings may inform policy development to scale-up the coverage of HTC amongst drug users, their sexual partners, and peers. We a high level of WTP for HTCs among MMT patient. Furthermore, almost half of respondents were willing to refer to their partners/relatives and more than one third of them were willing to be voluntary peer educators. Adjusting to other factors, providing HTC integrated with MMT sites appeared to facilitate HTC uptake and interest in referring to peers to HTC among drug using populations.

**HTC uptake**

Most of the respondents (94.2%) reported ever receiving HTCs. These result were around much higher than the rate of HTC uptake in general drug use population (28.0%) and other high risks populations such as female sex workers (38%) or men who have sex with men (39.4%) [^15^](#_ENREF_15). This findings can be explained by the fact that MMT patients in Vietnam were selective as the availability of services was still limited, and those who were in MMT may have had strong motivation and supports from their families. Our result was also higher than HTC uptake of MMT patients in China (75.7%) [^28^](#_ENREF_28), Indonesia (44%) [^47^](#_ENREF_47) and USA (34%) [^48^](#_ENREF_48). MMT has been shown to reduce frequency of HIV risk behavior and increase the use of HIV-related services . This study contributes to the literature by demonstrating that enrollment in MMT may empower patients to be catalysts for accelerating the expansion of HIV testing amongst at risk populations. In addition, previous research indicated that HIV testing results did not influence MMT retention but provided information for drug users to avoid transmitting HIV and early access to HIV care and treatment services [^50^](#_ENREF_50). Therefore, increasing the coverage of MMT program may have a significant role in expanding the coverage of HTC.

Patients participating in an integrative MMT-HTC clinic were found to have higher number of HTC visits compared to others. In some settings, providing HTC at MMT clinics may eliminate several barriers for uptake such as distance and lack of transports . Easy to access HTC promotes uptake among drug users and routine HIV testing is recommended in MMT clinics . However, protecting confidentiality in integrative HTC models should be addressed. Some studies illustrated that in this model, confidentiality might be at risk due to the lack of privacy, staff training, and power differentials between providers and clients [^53-56^](#_ENREF_53), while other surveys report opposite results .

**HTC referrals**

Since PWID sexual partners are at high risk of HIV infection , present study indicates the feasibility of MMT patients referring their sexual partners to HTC. A review of Hogben revealed that partner referral was an effective way to identify HIV-positive case[^61^](#_ENREF_61). However, in Vietnam, a study of Hong et al. showed that only 1.9% clients were referred by sexual partners and only one of four clients utilized HTC because their sexual partners were HIV-infected or in high-risk populations [^62^](#_ENREF_62). In our study, almost half of respondents were willing to refer partners/relatives to HTC and one third of sample was also willing to be voluntary peer educators. Those findings suggested a potential referral channel for promoting HTC among approach hidden populations.

Some facilitative factors for HTC referrals in this study were living with spouse, higher education, having white collar jobs, HIV-positive status, and greater number of HTC experiences. Indeed, those facilitators were frequently related with high level of knowledge and attitudes about HIV and HTC [^63-65^](#_ENREF_63); hence, those with greater knowledge may perceived the importance of HTC and were more willing to refer their partners/relatives. Moreover, people who used the integrative clinic model were more likely to express willingness to be voluntary peer educators. As demonstrated, integrative MMT-HTC clinics could improve HTC utilization. It may help patients to understand clearly the important role of HTC to prevent HIV transmission among drug use population, and then, encourage drug users to be voluntary peer educators.

Additionally, the results suggest that patients receiving referrals from peers for the first HTC use were more willing to be voluntary peer instructors. In HIV program, peer education is regularly used to prevent HIV infection and other sexual transmitted infections [^66^](#_ENREF_66). Peer educators had similar characteristics or behaviors with high risk population, therefore they may have high level of trust and comfort with their peers [^67^](#_ENREF_67). Moreover, they can more easily access hidden HIV population as compared to other approaches [^68^](#_ENREF_68). Thus, the role of peer educators is critical in encouraging HTC uptake and referrals of MMT patients.

Notably, having pain/discomfort was recognized as a barrier preventing the referral to relatives among MMT patients. Likewise, having mobility problem was negatively related with being voluntary peer instructors. The reason for this association is not be clear, but a study of Yang et al. from China showed that people having poor health status had more negative attitudes toward HTC than those having better health status [^69^](#_ENREF_69), thus they did not want to refer and to volunteer. Collectively, addressing health problems of MMT patients should be considered to promote HTC referrals among this population.

**Willingness to pay for HTC**

In this study, we observed an enormous proportion of patients willing to pay for HTC (91.6%) with the mean amount of US $17.9. Although this amount of WTP seems to be higher than the current user fees applied for HTC in Vietnam, it was about a half of the economic cost for the service in 2007 (US $38.9) [^33^](#_ENREF_33). This high level of willingness can be explained by the fact that our sample was primarily HIV-negative drug users who valued HTC and had strong motivation and familial supports to change their health behaviors. Consistent with previous studies, factors associated with WTP for HTC included older age and higher education and income . Furthermore, those who were willing to volunteer to be peer educator were also willing to pay more. In prior literatures, positive attitudes for a product demonstrates a strong association with WTP [^75-77^](#_ENREF_75). Conversely, people having usual-activities and pain/discomfort problems were observed to have lower amounts of WTP for HTC, which was consistent with previous studies that poor health status could be a negative factor of WTP .

**Implications**

This study had several implications. First, HTC should be integrated with MMT clinics to encourage testing and referrals among drug users and their sexual partners and peers. This integrative model could also help to reduce the duplicated operation cost by provider and transportation costs by users. Second, providing medical care promptly to patients having health problems is likely to improve their interest in providing referrals or being voluntary peer educators and increase willingness to pay for HTC. Third, peer educators in clinics are needed to promote patients’ HTC. Voluntary peer instructors may be effective by sharing experience and encouraging participating in peer educator groups. Those factors mentioned above could help to effectively expand HTC to other at risk populations. Future studies may be useful to assess if relatives and partners of MMT patients have greater awareness on HIV/AIDS, maintain healthy behaviors, and engage in HTC. Finally, the result suggest that resources mobilization through users’ fees for VCT may be applied to ensure the sustainability of HIV/AIDS program. During the study period, co-payment for HTC has been piloted in some HTC clinics in Vietnam with US $1.5 to 2.5 per visit. The amount that patients were willing to pay for HTC in this paper was hypothetical. Further translational research is needed on actual WTP before becoming the basis for HTC policy. Also, it is important to note that the application of user fee for HIV testing could serve as a barrier for the most vulnerable and highest risk segment of the MMT population. Our findings suggest that the justification of user fee can be based on economic cost analyses with continuous subsidy for socioeconomically disadvantaged groups. Additionally, due to the fact that the cost per HTC uptake is depend on the number of clients, program managers should use performance-based incentives for peer instructors to refer targeted populations to HTC [^32^](#_ENREF_32).

**Limitations**

This study has several limitations. First, recall bias may be occurred in self-reported data. Second, the sensitive questions about drug use or sexual behaviors be subject to social desirability bias. Furthermore, cross-sectional study may limit the establishment of causal relations between HTC uptake, referral, willingness to pay and MMT treatment. Finally, the convenient sample may limit generalizability of the findings to a larger population.

**Conclusions**

In conclusion, this study showed a high willingness of MMT patients to pay for HTC. Moreover, about a half of the patients were willing to refer their partners and relatives to HTC services, and one third to be voluntary peer instructors. Integrating HIV testing with MMT services and applying user’s fee are potential strategies to mobilize resources and encourage HIV testing among MMT patients and their partners.

**TABLES AND FIGURES**

Table 1: Study settings and sample size

| **Level** | **Settings** | **Site Name** | **Type of services** | **Sample size** |
| --- | --- | --- | --- | --- |
| Province | Nam Dinh City | Provincial AIDS Center | MMT+ HTC | 270 |
| District (rural) | Xuan Truong District | District Health Center | MMT+ HTC + ART + GH | 151 |
| District (urban) | Tu Liem District | District Health Center | MMT+ HTC + ART + GH | 201 |
| District (urban) | Long Bien District | District Health Center | MMT+ HTC + ART + GH | 184 |
| District (urban) | Ha Dong District | Regional Polyclinic | MMT+ GH | 210 |

**Table 2: Demographics and health-related quality of life** of respondents

|  | **Without HTC** | | **With HTC** | | **Total** | | **p-value** |
| --- | --- | --- | --- | --- | --- | --- | --- |
|  | **N** | **%** | **N** | **%** | **N** | **%** |  |
| **Age** |  |  |  |  |  |  |  |
| 18-<25 | 7 | 3.3 | 14 | 1.7 | 21 | 2.1 | 0.14 |
| 25-<30 | 33 | 15.7 | 106 | 13.2 | 139 | 13.7 |  |
| 30- <35 | 53 | 25.2 | 213 | 26.4 | 266 | 26.2 |  |
| 35- <40 | 43 | 20.5 | 223 | 27.7 | 266 | 26.2 |  |
| 40- <45 | 42 | 20.0 | 125 | 15.5 | 167 | 16.4 |  |
| >=45 | 32 | 15.2 | 125 | 15.5 | 157 | 15.5 |  |
| **Marital status** |  |  |  |  |  |  |  |
| Single | 47 | 22.4 | 204 | 25.3 | 251 | 24.7 | 0.66 |
| Live with spouse | 147 | 70.0 | 538 | 66.8 | 685 | 67.4 |  |
| Live with partner | 1 | 0.5 | 2 | 0.3 | 3 | 0.3 |  |
| Divorced | 15 | 7.1 | 57 | 7.1 | 72 | 7.1 |  |
| Widow | 0 | 0.0 | 5 | 0.6 | 5 | 0.5 |  |
| **Educational attainment** |  |  |  |  |  |  |  |
| Illiterate | 4 | 1.9 | 13 | 1.6 | 17 | 1.7 | 0.93 |
| Elementary | 27 | 12.9 | 92 | 11.4 | 119 | 11.7 |  |
| Secondary | 86 | 41.0 | 340 | 42.2 | 426 | 41.9 |  |
| High | 81 | 38.6 | 306 | 38.0 | 387 | 38.1 |  |
| Vocational | 7 | 3.3 | 25 | 3.1 | 32 | 3.2 |  |
| University | 5 | 2.4 | 30 | 3.7 | 35 | 3.4 |  |
| **Employment** |  |  |  |  |  |  |  |
| Unemployed | 53 | 25.2 | 206 | 25.6 | 259 | 25.5 | 0.89 |
| Self-employed | 112 | 53.3 | 430 | 53.4 | 542 | 53.4 |  |
| White collars | 5 | 2.4 | 17 | 2.1 | 22 | 2.2 |  |
| Workers, Farmers | 18 | 8.6 | 82 | 10.2 | 100 | 9.8 |  |
| Students |  |  | 2 | 0.3 | 2 | 0.2 |  |
| Other jobs | 22 | 10.5 | 69 | 8.6 | 91 | 9.0 |  |
| **Religion** |  |  |  |  |  |  |  |
| Cult of ancestors | 198 | 94.3 | 698 | 86.6 | 896 | 88.2 | <0.05 |
| Buddhism | 10 | 4.8 | 49 | 6.1 | 59 | 5.8 |  |
| Catholic | 2 | 1.0 | 54 | 6.7 | 56 | 5.5 |  |
| Protestant | 0 | 0.0 | 5 | 0.7 | 5 | 0.5 |  |
| **Health status** |  |  |  |  |  |  |  |
| Having mobility problem | 15 | 7.1 | 59 | 7.3 | 74 | 7.3 | 0.93 |
| Having self-care problem | 9 | 4.3 | 31 | 3.9 | 40 | 3.9 | 0.77 |
| Having usual activities problem | 9 | 4.3 | 51 | 6.3 | 60 | 5.9 | 0.26 |
| Having pain/discomfort | 34 | 16.2 | 146 | 18.1 | 180 | 17.7 | 0.52 |
| Having anxiety/depression | 38 | 18.1 | 172 | 21.3 | 210 | 20.7 | 0.30 |

**Table 3: Sexual behaviors among respondents**

|  | **Without HTC** | | **With HTC** | | **Total** | | **p-value** |
| --- | --- | --- | --- | --- | --- | --- | --- |
|  | **N** | **%** | **N** | **%** | **N** | **%** |
| **Ever had sex** | 210 | 100.0 | 794 | 98.5 | 1,004 | 98.8 | 0.07 |
| **Number of sexual partners (in the last 12 months)** |  |  |  |  |  |  |  |
| Not had anyone | 24 | 11.4 | 159 | 19.7 | 183 | 18.0 | <0.05 |
| One sex partners | 156 | 74.3 | 552 | 68.5 | 708 | 69.7 |  |
| 2-3 sex partners | 13 | 6.2 | 33 | 4.1 | 46 | 4.5 |  |
| >4 sex partners | 17 | 8.1 | 62 | 7.7 | 79 | 7.8 |  |
| **Type of sex partner** |  |  |  |  |  |  |  |
| Primary partners | 172 | 81.9 | 628 | 77.9 | 800 | 78.7 | 0.21 |
| Casual sex partners | 13 | 6.2 | 48 | 6.0 | 61 | 6.0 | 0.90 |
| Sex workers | 12 | 5.7 | 70 | 8.7 | 82 | 8.1 | 0.16 |
| **Inconsistent condom use** |  |  |  |  |  |  |  |
| With Primary sexual partners (n=800) | 142 | 82.6 | 433 | 69.0 | 575 | 71.9 | <0.001 |
| With Casual sexual partners (n=61) | 9 | 69.2 | 17 | 35.4 | 26 | 42.6 | <0.05 |
| With Sex workers (n=82) | 1 | 8.3 | 12 | 17.1 | 13 | 15.9 | 0.44 |
| **Percentage of condom use (in the last 12 months)** | **Mean** | **SD** | **Mean** | **SD** | **Mean** | **SD** |  |
| With Primary sex partners | 16.0 | 33.1 | 26.5 | 40.5 | 24.2 | 39.3 | <0.001 |
| With Casual sexual partners | 20.8 | 40.1 | 29.2 | 45.9 | 27.4 | 44.6 | 0.28 |
| With Sex workers | 33.3 | 49.2 | 30.7 | 45.9 | 86.4 | 33.2 | 0.43 |

**Table 4: Drug use behaviors among respondents**

| **Characteristics** | **Without HTC** | |  | **With HTC** | |  | **Total** | |  | **p-value** |
| --- | --- | --- | --- | --- | --- | --- | --- | --- | --- | --- |
|  | **N** | **%** |  | **N** | **%** |  | **N** | **%** |  |  |
| **Ever inject drug** | 161 | 76.7 |  | 585 | 72.6 |  | 746 | 73.4 |  | 0.23 |
| **Current drug use** | 11 | 5.2 |  | 38 | 4.7 |  | 49 | 4.8 |  | 0.75 |
| **# drug rehabilitation** |  |  |  |  |  |  |  |  |  |  |
| None | 18 | 8.6 |  | 56 | 7.0 |  | 74 | 7.3 |  | 0.09 |
| 1--5 episodes | 151 | 71.9 |  | 524 | 65.0 |  | 675 | 66.4 |  |  |
| 6--10 | 33 | 15.7 |  | 178 | 22.1 |  | 211 | 20.8 |  |  |
| >10 | 8 | 3.8 |  | 48 | 6.0 |  | 56 | 5.5 |  |  |
| **Location of previous drug rehabilitation** |  |  |  |  |  |  |  |  |  |  |
| Home | 141 | 67.1 |  | 545 | 70.9 |  | 686 | 70.1 |  | 0.30 |
| Private voluntary center | 99 | 47.1 |  | 354 | 46.2 |  | 453 | 46.4 |  | 0.80 |
| Compulsory center | 33 | 15.7 |  | 224 | 29.2 |  | 257 | 26.3 |  | <0.001 |
| **Reason for relapsed** |  |  |  |  |  |  |  |  |  |  |
| Boredom | 81 | 38.6 |  | 285 | 35.4 |  | 366 | 36.0 |  | 0.39 |
| Peer inducement | 113 | 53.8 |  | 372 | 46.2 |  | 485 | 47.7 |  | 0.05 |
| Craving | 99 | 47.1 |  | 340 | 42.2 |  | 439 | 43.2 |  | 0.20 |
| Unemployment | 6 | 2.9 |  | 43 | 5.3 |  | 49 | 4.8 |  | 0.14 |
| **HIV positive** | 9 | 4.3 |  | 73 | 9.1 |  | 82 | 8.1 |  | <0.05 |
| **ART** | 6 | 2.9 |  | 60 | 7.4 |  | 66 | 6.5 |  | <0.05 |
|  | **Mean** | **95% CI** | | **Mean** | **95% CI** | | **Mean** | **95% CI** | |  |
| **# previous drug rehabilitation episodes** | 4.6 | 3.9 | 5.4 | 5.5 | 5.0 | 6.1 | 5.3 | 4.9 | 5.8 | <0.05 |
| **Age at first drug use** | 25.3 | 24.4 | 26.2 | 24.3 | 23.9 | 24.8 | 24.5 | 24.1 | 25.0 | <0.05 |
| **Age at first drug injection** | 27.9 | 26.8 | 29.0 | 26.6 | 26.0 | 27.2 | 26.8 | 26.3 | 27.4 | <0.05 |
| **Time since 1st drug use (years)** | 12.2 | 11.4 | 13.0 | 13.6 | 13.2 | 14.0 | 13.3 | 12.9 | 13.6 | <0.05 |
| **Time since first drug injection (years)** | 9.2 | 8.5 | 9.9 | 10.5 | 10.1 | 10.9 | 10.2 | 9.9 | 10.6 | <0.05 |
| **Daily cost of drug use (1000 vnd)** | 356.4 | 244.7 | 468.1 | 322.3 | 277.5 | 367.1 | 326.8 | 285.4 | 368.2 | 0.29 |
| **Duration on MMT (month)** | 15.2 | 14.1 | 16.3 | 16.9 | 16.1 | 17.8 | 16.6 | 15.9 | 17.3 | <0.001 |

**Table 5: HTC uptake and willingness to pay among respondents**

| **Characteristics** | **Without HTC** | | | **With HTC** | | | **Total** | | | **p-value** |
| --- | --- | --- | --- | --- | --- | --- | --- | --- | --- | --- |
|  | **n** | **%** |  | **n** | **%** |  | **n** | **%** |  |  |
| **Ever use HTC** | 206 | 98.1 |  | 751 | 93.2 |  | 957 | 94.2 |  | <0.05 |
| **Referrer of the first HTC used** |  |  |  |  |  |  |  |  |  |  |
| Spouse | 4 | 1.9 |  | 24 | 3.2 |  | 28 | 2.9 |  | <0.05 |
| Peers | 5 | 2.4 |  | 33 | 4.4 |  | 38 | 4.0 |  |  |
| Health workers | 149 | 72.3 |  | 421 | 56.1 |  | 570 | 59.6 |  |  |
| Media | 1 | 0.5 |  | 12 | 1.6 |  | 13 | 1.4 |  |  |
| Self-motivation | 42 | 20.4 |  | 226 | 30.1 |  | 268 | 28.0 |  |  |
| Parents/Relatives | 5 | 2.4 |  | 35 | 4.7 |  | 40 | 4.2 |  |  |
| **HIV status of relatives (Positive)** |  |  |  |  |  |  |  |  |  |  |
| Family members | 6 | 2.9 |  | 40 | 5.0 |  | 46 | 4.5 |  | 0.19 |
| Spouse/Partners | 2 | 1.0 |  | 3 | 0.4 |  | 5 | 0.5 |  | 0.28 |
| Parents | 0 | 0.0 |  | 1 | 0.1 |  | 1 | 0.1 |  | 0.61 |
| Brother/Sister | 2 | 1.0 |  | 4 | 0.5 |  | 6 | 0.6 |  | 0.44 |
| Kinship | 0 | 0.0 |  | 5 | 0.6 |  | 5 | 0.5 |  | 0.25 |
| Other relatives | 1 | 0.5 |  | 0 | 0.0 |  | 1 | 0.1 |  | 0.05 |
| **Refer partners to HIV testing services** | 85 | 40.5 |  | 379 | 47.0 |  | 464 | 45.7 |  | 0.1 |
| **Refer other relatives to HIV testing services** | 70 | 33.3 |  | 289 | 35.9 |  | 359 | 35.3 |  | 0.5 |
| **Volunteer to be a Peer educator** | 52 | 24.8 |  | 286 | 35.5 |  | 338 | 33.3 |  | <0.05 |
| **Willing to pay for HTC** | 188 | 89.5 |  | 743 | 92.2 |  | 931 | 91.6 |  | 0.22 |
|  | **Mean** | **95% CI** | | **Mean** | **95% CI** | | **Mean** | **95% CI** | |  |
| **# HTC test uptake** | 4.3 | 0.7 | 8.0 | 6.8 | 5.8 | 7.9 | 6.6 | 5.6 | 7.6 | 0.09 |
| **Willingness to pay for a HTC (thousand VND)** | 304 | 252 | 355 | 373 | 343 | 403 | 358 | 332 | 385 | 0.04 |

**Table 6: Factors associated with the use of and WTP for HTC**s and referrals for sexual partners and other relatives among MMT patients

| **Characteristics** | **Willingness to pay for HTC** | | | **# HIV test uptake** | | | **Refer partners to HTD** | | | **Refer other relatives to HTC** | | | **Volunteer to be a Peer educator** | | |
| --- | --- | --- | --- | --- | --- | --- | --- | --- | --- | --- | --- | --- | --- | --- | --- |
|  | **Coef** | **95% CI** | | **Coef** | **95% CI** | | **OR** | **95% CI** | | **OR** | **95% CI** | | **OR** | **95% CI** | |
| **Sex (Female vs. Male)** |  |  |  |  |  |  |  |  |  | 2.4 | 0.7 | 8.5 |  |  |  |
| **Age (**18- <25 - ref) |  |  |  |  |  |  |  |  |  |  |  |  |  |  |  |
| 25- <30 |  |  |  |  |  |  |  |  |  |  |  |  | 0.3* | 0.1 | 0.8 |
| 30- <35 |  |  |  |  |  |  |  |  |  | 0.8 | 0.5 | 1.0 | 0.3* | 0.1 | 0.9 |
| 35- <40 |  |  |  | 0.3 | -0.1 | 0.7 |  |  |  |  |  |  | 0.3* | 0.1 | 0.8 |
| 40- <45 | 82.6* | 10.8 | 154.3 |  |  |  |  |  |  |  |  |  | 0.4 | 0.1 | 1.2 |
| >=45 |  |  |  |  |  |  |  |  |  | 0.7 | 0.5 | 1.1 | 0.2* | 0.1 | 0.6 |
| **Marital status** (Single-ref) |  |  |  |  |  |  |  |  |  |  |  |  |  |  |  |
| Living with spouse |  |  |  | -0.5* | -1.0 | 0.0 | 1.6* | 1.1 | 2.3 |  |  |  |  |  |  |
| Divorced |  |  |  |  |  |  |  |  |  |  |  |  |  |  |  |
| Widow |  |  |  | 2.9* | 0.2 | 5.6 | 1.8 | 1.0 | 3.3 | 9.7 | 0.9 | 100.6 |  |  |  |
| **Religion** (Cult of ancestors – ref) |  |  |  |  |  |  | 11.5 | 1.0 | 130.0 |  |  |  |  |  |  |
| Catholic |  |  |  | -0.8 | -1.6 | 0.1 |  |  |  |  |  |  |  |  |  |
| Protestant |  |  |  |  |  |  |  |  |  |  |  |  | 5.8 | 0.6 | 52.8 |
| **Education** (Illiterate – ref) |  |  |  |  |  |  |  |  |  |  |  |  |  |  |  |
| Elementary | 103.9* | 15.0 | 192.8 |  |  |  |  |  |  | 0.6 | 0.4 | 1.0 | 0.6* | 0.4 | 1.0 |
| Secondary | 84.9* | 27.7 | 142.0 |  |  |  | 1.5* | 1.0 | 2.2 |  |  |  |  |  |  |
| Vocational | 210.2* | 55.4 | 365.0 |  |  |  | 1.5* | 1.0 | 2.3 |  |  |  |  |  |  |
| University |  |  |  | -0.7 | -1.8 | 0.4 |  |  |  |  |  |  |  |  |  |
| **Employment** (Unemployed – ref) |  |  |  |  |  |  |  |  |  |  |  |  |  |  |  |
| Self-employed |  |  |  |  |  |  | 1.4 | 1.0 | 1.8 | 1.3 | 0.9 | 1.7 |  |  |  |
| White collars | 127.1 | -51.3 | 305.5 |  |  |  | 2.9* | 1.1 | 7.9 | 2.8* | 1.1 | 7.0 | 1.8 | 0.7 | 4.5 |
| Other jobs |  |  |  | 0.8* | 0.1 | 1.5 |  |  |  | 0.7 | 0.4 | 1.2 |  |  |  |
| **Income per capita** (Poorest – ref) |  |  |  |  |  |  |  |  |  |  |  |  |  |  |  |
| Poor |  |  |  | 0.4 | -0.2 | 0.9 | 0.8 | 0.5 | 1.1 |  |  |  |  |  |  |
| Middle | 80.9* | 12.5 | 149.4 | 0.4 | -0.2 | 0.9 | 0.7 | 0.5 | 1.0 |  |  |  |  |  |  |
| Rich | 98.3* | 31.3 | 165.3 | 0.7* | 0.2 | 1.3 |  |  |  |  |  |  |  |  |  |
| **HIV status (Positive vs. Negative)** |  |  |  | 1.2* | 0.5 | 1.9 | 2.3* | 1.3 | 4.0 | 2.9* | 1.7 | 4.9 |  |  |  |
| **Kinship HIV status (Positive vs. Negative)** |  |  |  |  |  |  |  |  |  | 5.2 | 0.7 | 38.0 |  |  |  |
| **Mobility (Have problems vs. No problems)** |  |  |  |  |  |  |  |  |  |  |  |  | 0.4* | 0.2 | 0.7 |
| **Self-care (Have problems vs. No problems)** |  |  |  |  |  |  |  |  |  | 2.0 | 0.8 | 4.7 |  |  |  |
| **Usual Activities (Have problems vs. No)** | -118.8* | -234.2 | -3.5 |  |  |  |  |  |  | 0.5 | 0.2 | 1.1 | 2.8 | 1.4 | 5.8 |
| **Pain/Discomfort (Have problems vs. No)** | -108.1* | -181.4 | -34.7 |  |  |  | 0.7 | 0.5 | 1.0 | 0.6* | 0.4 | 0.9 | 0.7 | 0.4 | 1.0 |
| **MMT service model (with HTC vs. without HTC)** | 45.3 | -19.6 | 110.2 | 0.6* | 0.1 | 1.1 |  |  |  |  |  |  | 1.9* | 1.3 | 2.8 |
| **Referrer of the first HTC used** (Spouse-ref) |  |  |  |  |  |  |  |  |  |  |  |  |  |  |  |
| Peers |  |  |  | 0.9 | -0.1 | 1.8 |  |  |  |  |  |  | 2.7* | 1.4 | 5.5 |
| Health workers | -105.2* | -187.6 | -22.7 |  |  |  | 0.5* | 0.3 | 0.8 |  |  |  |  |  |  |
| Media |  |  |  | 1.2 | -0.4 | 2.9 |  |  |  |  |  |  | 2.3 | 0.7 | 7.2 |
| Self-motivation | -60.2 | -149.5 | 29.1 | 0.4* | 0.0 | 0.9 | 0.7 | 0.4 | 1.1 | 1.3 | 0.9 | 1.8 |  |  |  |
| **Current drug use (Yes vs. No)** | 86.1 | -41.8 | 214.1 |  |  |  | 0.5 | 0.3 | 1.1 |  |  |  |  |  |  |
| **Condom use with primary partner (**Yes-ref) |  |  |  |  |  |  |  |  |  |  |  |  |  |  |  |
| No |  |  |  |  |  |  | 0.7 | 0.5 | 1.0 | 0.8 | 0.5 | 1.1 |  |  |  |
| Not have sexual intercourse |  |  |  | -0.5 | -1.1 | 0.1 | 0.4 | 0.2 | 0.6 | 0.5* | 0.4 | 0.8 |  |  |  |
| **Condom use with Casual sexual partners (**Yes-ref) |  |  |  |  |  |  |  |  |  |  |  |  |  |  |  |
| No |  |  |  |  |  |  |  |  |  | 5.5 | 0.4 | 69.2 |  |  |  |
| Not have sexual intercourse |  |  |  |  |  |  |  |  |  | 1.7 | 0.9 | 3.2 |  |  |  |
| **Times taking HTC** |  |  |  |  |  |  | 1.1* | 1.1 | 1.2 | 1.1* | 1.0 | 1.1 |  |  |  |
| **Refer partners to HTC (Yes vs. No)** |  |  |  | 1.0* | 0.6 | 1.4 |  |  |  |  |  |  |  |  |  |
| **Refer other relatives to HTC (Yes vs. No)** | -57.8 | -115.9 | 0.2 |  |  |  |  |  |  |  |  |  |  |  |  |
| **Volunteer to be a Peer educator (Yes vs. No)** | 128.8* | 68.9 | 188.8 | -0.4 | -0.8 | 0.0 |  |  |  |  |  |  |  |  |  |
| **Constant** | 297.6 | 188.4 | 406.9 | 2.3 | 1.6 | 3.0 | 0.7 | 0.4 | 1.4 | 0.3 | 0.2 | 0.7 | 1.1 | 0.4 | 3.2 |

**References**

**1.** The Commission on AIDS in Asia. Redefining AIDS in Asia: Crafting an effective response. *Oxford University Press. 258 pages. Available at http://www.unaids.org/en/media/unaids/contentassets/dataimport/pub/report/2008/20080326_report_commission_aids_en.pdf. Accessed November 1, 2012.* 2008.

**2.** WHO, UNAIDS, UNICEF. *Towards universal access: Scaling up priority HIV/AIDS interventions in the health sector.* Geneva, Switzerland: WHO, UNAIDS, and UNICEF;2009.

**3.** HIV/AIDS JUNPo. *90-90-90: An ambitious treatment target to help end the AIDS epidemic*: UNAIDS;2014.

**4.** Organization WH. *Consolidated guidelines on HIV testing services 2015.* Geneva, Switzerland: World Health Organization;2015.

**5.** Health Mo. Decision on promulgation of voluntary HIV counselling and testing (VCT) guidelines. Hanoi2007.

**6.** Fonner VA, Denison J, Kennedy CE, O’Reilly K, Sweat M. Voluntary counseling and testing (VCT) for changing HIV-related risk behavior in developing countries. *Cochrane database of systematic reviews.*9:Cd001224.

**7.** Matovu JK, Gray RH, Makumbi F, et al. Voluntary HIV counseling and testing acceptance, sexual risk behavior and HIV incidence in Rakai, Uganda. *Aids.* Mar 25 2005;19(5):503-511.

**8.** Corbett EL, Makamure B, Cheung YB, et al. HIV incidence during a cluster-randomized trial of two strategies providing voluntary counselling and testing at the workplace, Zimbabwe. *Aids.* Feb 19 2007;21(4):483-489.

**9.** Organization WH. *Service delivery approaches to HIV testing and counselling (HTC): a strategic HTC policy framework.* Geneva, Switzerland: World Health Organization;2012.

**10.** National Committee for AIDS DaPPaC. National Strategy on HIV/AIDS Prevention and Control toward 2020 and the vision to 2030. Hanoi2012.

**11.** Tran BX, Nguyen LT, Nguyen NP, Phan HT. HIV voluntary testing and perceived risk among female sex workers in the Mekong Delta region of Vietnam. *Global health action.* 2013;6:20690.

**12.** Control VAoHA. *The annual review of HIV/AIDS control and prevention in the first six months 2015 and action plan in the last six months in 2015.* Hanoi: Ministry of Health;2015.

**13.** Nguyen TA, Oosterhoff P, Hardon A, Tran HN, Coutinho RA, Wright P. A hidden HIV epidemic among women in Vietnam. *BMC public health.* 2008;8:37.

**14.** Control VAoHA. *Vietnam HIV/AIDS Estimates and Projections 2011 - 2015.* Hanoi: Ministry of Health;2013.

**15.** Son VH. Strategy on HIV counseling and testing during 2016-2020. *Vietnam National AIDS Conference 2015*. Hanoi, Vietnam2015.

**16.** Tran BX. Willingness to pay for methadone maintenance treatment in Vietnamese epicentres of injection-drug-driven HIV infection. *Bulletin of the World Health Organization.* Jul 1 2013;91(7):475-482.

**17.** Tran BX, Ohinmaa A, Duong AT, et al. The cost-effectiveness and budget impact of Vietnam's methadone maintenance treatment programme in HIV prevention and treatment among injection drug users. *Global public health.* 2012;7(10):1080-1094.

**18.** Nguyen TT, Nguyen LT, Pham MD, Vu HH, Mulvey KP. Methadone maintenance therapy in Vietnam: an overview and scaling-up plan. *Advances in preventive medicine.* 2012;2012:732484.

**19.** Gowing L, Farrell M, Bornemann R, Sullivan L, Ali R. Substitution treatment of injecting opioid users for prevention of HIV infection. *The Cochrane database of systematic reviews.* 2008(2):CD004145.

**20.** MacArthur GJ, Minozzi S, Martin N, et al. Opiate substitution treatment and HIV transmission in people who inject drugs: systematic review and meta-analysis. *Bmj.* 2012;345:e5945.

**21.** Tran BX, Ohinmaa A, Duong AT, et al. Changes in drug use are associated with health-related quality of life improvements among methadone maintenance patients with HIV/AIDS. *Qual Life Res.* May 2012;21(4):613-623.

**22.** Zhang L, Chow EP, Zhuang X, et al. Methadone maintenance treatment participant retention and behavioural effectiveness in China: a systematic review and meta-analysis. *PloS one.* 2013;8(7):e68906.

**23.** Wang M, Mao W, Zhang L, et al. Methadone maintenance therapy and HIV counseling and testing are associated with lower frequency of risky behaviors among injection drug users in China. *Substance use & misuse.* Jan 2015;50(1):15-23.

**24.** Tran BX, Ohinmaa A, Mills S, et al. Multilevel predictors of concurrent opioid use during methadone maintenance treatment among drug users with HIV/AIDS. *PloS one.* 2012;7(12):e51569.

**25.** Tran BX, Nguyen TV, Pham QD, et al. HIV infection, risk factors, and preventive services utilization among female sex workers in the Mekong Delta Region of Vietnam. *PloS one.* 2014;9(1):e86267.

**26.** Nguyen LH, Tran BX, Nguyen NP, Phan HT, Bui TT, Latkin CA. Mobilization for HIV Voluntary Counseling and Testing Services in Vietnam: Clients' Risk Behaviors, Attitudes and Willingness to Pay. *AIDS and behavior.* Sep 12 2015.

**27.** Markwick N, Ti L, Callon C, Feng C, Wood E, Kerr T. Willingness to engage in peer-delivered HIV voluntary counselling and testing among people who inject drugs in a Canadian setting. *Journal of epidemiology and community health.* July 1, 2014 2014;68(7):675-678.

**28.** Xia YH, Chen W, Tucker JD, Wang C, Ling L. HIV and hepatitis C virus test uptake at methadone clinics in Southern China: opportunities for expanding detection of bloodborne infections. *BMC Public Health.* 2013;13:899.

**29.** Day CA, White B, Thein HH, et al. Experience of hepatitis C testing among injecting drug users in Sydney, Australia. *AIDS Care.* Jan 2008;20(1):116-123.

**30.** UNAIDS. *Vietnam National AIDS Spending Assessment 2011–2012.* Hanoi, Vietnam: UNAIDS;2014.

**31.** Tran BX, Duong AT, Nguyen LT, et al. Financial burden of health care for HIV/AIDS patients in Vietnam. *Trop Med Int Health.* Feb 2013;18(2):212-218.

**32.** Nguyen VT, Nguyen HT, Nguyen QC, Duong PT, West G. Expenditure Analysis of HIV Testing and Counseling Services Using the Cascade Framework in Vietnam. *PloS one.* 2015;10(5):e0126659.

**33.** Minh HV, Bach TX, Mai NY, Wright P. The cost of providing HIV/AIDS counseling and testing services in Vietnam *Valua in Health regional.* 2012(1):36-40.

**34.** Minister P. Ensuring finance for HIV/AIDS prevention and control activities in the period 2013-2020 In: Health Mo, ed. *1899/QD-TTg*2013.

**35.** Metsch L, Philbin MM, Parish C, Shiu K, Frimpong JA, Giang le M. HIV Testing, Care, and Treatment Among Women Who Use Drugs From a Global Perspective: Progress and Challenges. *J Acquir Immune Defic Syndr.* Jun 1 2015;69 Suppl 2:S162-168.

**36.** Tran BX, Nguyen LH, Phan HT, Nguyen LK, Latkin CA. Preference of methadone maintenance patients for the integrative and decentralized service delivery models in Vietnam. *Harm reduction journal.* 2015;12(1):29.

**37.** Tran BX, Nguyen LH, Phan HT, Nguyen LK, Latkin CA. Preference of methadone maintenance patients for the integrative and decentralized service delivery models in Vietnam. *Harm reduction journal.* 2015;12:29.

**38.** Tran BX, Nguyen LH, Phan HT, Latkin CA. Patient Satisfaction with Methadone Maintenance Treatment in Vietnam: A Comparison of Different Integrative-Service Delivery Models. *PloS one.* 2015;10(11):e0142644.

**39.** Tran BX, Nguyen LH, Do HP, et al. Motivation for smoking cessation among drug-using smokers under methadone maintenance treatment in Vietnam. *Harm reduction journal.* 2015;12(1):50.

**40.** Group E. EQ-5D-5L User Guide: Basic information on how to use the EQ-5D-5L instrument. 2011; http://www.euroqol.org/fileadmin/user_upload/Documenten/PDF/Folders_Flyers/UserGuide_EQ-5D-5L.pdf. Accessed 1-9, 2013.

**41.** Tran BX, Ohinmaa A, Nguyen LT, Nguyen TA, Nguyen TH. Determinants of health-related quality of life in adults living with HIV in Vietnam. *AIDS Care.* Oct 2011;23(10):1236-1245.

**42.** Tran BX, Houston S. Mobile phone-based antiretroviral adherence support in Vietnam: feasibility, patient's preference, and willingness-to-pay. *AIDS and behavior.* Oct 2012;16(7):1988-1992.

**43.** Tran BX, Ohinmaa A, Nguyen LT. Quality of life profile and psychometric properties of the EQ-5D-5L in HIV/AIDS patients. *Health Qual Life Outcomes.* 2012;10:132.

**44.** Tran BX, Nguyen LT. Impact of methadone maintenance on health utility, health care utilization and expenditure in drug users with HIV/AIDS. *The International journal on drug policy.* Nov 2013;24(6):e105-110.

**45.** Tran B, Nguyen L, Ohinmaa A, Maher R, Nong V, Latkin CA. Longitudinal and cross sectional assessments of health utility in adults with HIV/AIDS: a systematic review and meta-analysis. *BMC Health Serv Res.* Jan 22 2015;15(1):7.

**46.** Herriges JA, Shogren JF. Starting Point Bias in Dichotomous Choice Valuation with Follow-Up Questioning. *Journal of Environmental Economics and Management.* 1// 1996;30(1):112-131.

**47.** Achmad YM, Istiqomah AN, Iskandar S, Wisaksana R, van Crevel R, Hidayat T. Integration of methadone maintenance treatment and HIV care for injecting drug users: a cohort study in Bandung, Indonesia. *Acta medica Indonesiana.* Jul 2009;41 Suppl 1:23-27.

**48.** Seewald R, Bruce RD, Elam R, et al. Effectiveness and feasibility study of routine HIV rapid testing in an urban methadone maintenance treatment program. *Am J Drug Alcohol Abuse.* Jul 2013;39(4):247-251.

**49.** Ward J, Mattick RP, Hall W. The effectiveness of methadone maintenance treatment: an overview. *Drug and alcohol review.* 1994;13(3):327-335.

**50.** Wimbush J, Amicarelli A, Stein MD. Does HIV test result influence methadone maintenance treatment retention? *Journal of substance abuse.* 1996;8(2):263-269.

**51.** Ma W, Detels R, Feng Y, et al. Acceptance of and barriers to voluntary HIV counselling and testing among adults in Guizhou province, China. *Aids.* Dec 2007;21 Suppl 8:S129-135.

**52.** van Dyk AC, van Dyk PJ. "To know or not to know": service-related barriers to voluntary HIV counseling and testing (VCT) in South Africa. *Curationis.* May 2003;26(1):4-10.

**53.** Rennie S, Behets F. Desperately seeking targets: the ethics of routine HIV testing in low-income countries. *Bulletin of the World Health Organization.* Jan 2006;84(1):52-57.

**54.** McQuoid-Mason D. Routine testing for HIV--ethical and legal implications. *South African medical journal = Suid-Afrikaanse tydskrif vir geneeskunde.* Jun 2007;97(6):416, 418, 420.

**55.** Becker J, Tsague L, Sahabo R, Twyman P. Provider Initiated Testing and Counseling (PITC) for HIV in resource-limited clinical settings: important questions unanswered. *The Pan African medical journal.* 2009;3:4.

**56.** Corneli A, Jarrett NM, Sabue M, et al. Patient and provider perspectives on implementation models of HIV counseling and testing for patients with TB. *The international journal of tuberculosis and lung disease : the official journal of the International Union against Tuberculosis and Lung Disease.* Mar 2008;12(3 Suppl 1):79-84.

**57.** Obermeyer CM, Neuman M, Desclaux A, et al. Associations between mode of HIV testing and consent, confidentiality, and referral: a comparative analysis in four African countries. *PLoS medicine.* 2012;9(10):e1001329.

**58.** Weiser SD, Heisler M, Leiter K, et al. Routine HIV testing in Botswana: a population-based study on attitudes, practices, and human rights concerns. *PLoS medicine.* Jul 2006;3(7):e261.

**59.** Panda S, Chatterjee A, Bhattacharya SK, et al. Transmission of HIV from injecting drug users to their wives in India. *Int J STD AIDS.* Jul 2000;11(7):468-473.

**60.** Saxon AJ, Calsyn DA, Whittaker S, Freeman G, Jr. Sexual behaviors of intravenous drug users in treatment. *J Acquir Immune Defic Syndr.* 1991;4(10):938-944.

**61.** Hogben M, McNally T, McPheeters M, Hutchinson AB. The effectiveness of HIV partner counseling and referral services in increasing identification of HIV-positive individuals a systematic review. *American journal of preventive medicine.* Aug 2007;33(2 Suppl):S89-100.

**62.** Hong NT, Wolfe MI, Dat TT, et al. Utilization of HIV voluntary counseling and testing in Vietnam: an evaluation of 5 years of routine program data for national response. *AIDS education and prevention : official publication of the International Society for AIDS Education.* Jun 2011;23(3 Suppl):30-48.

**63.** Mahmoud MM, Nasr AM, Gassmelseed DE, Abdalelhafiz MA, Elsheikh MA, Adam I. Knowledge and attitude toward HIV voluntary counseling and testing services among pregnant women attending an antenatal clinic in Sudan. *Journal of medical virology.* May 2007;79(5):469-473.

**64.** Iliyasu Z, Abubakar IS, Kabir M, Aliyu MH. Knowledge of HIV/AIDS and attitude towards voluntary counseling and testing among adults. *Journal of the National Medical Association.* Dec 2006;98(12):1917-1922.

**65.** Hutchinson PL, Mahlalela X. Utilization of voluntary counseling and testing services in the Eastern Cape, South Africa. *AIDS Care.* Jul 2006;18(5):446-455.

**66.** Medley A, Kennedy C, O’Reilly K, Sweat M. Effectiveness of Peer Education Interventions for HIV Prevention in Developing Countries: A Systematic Review and Meta-Analysis. *AIDS education and prevention : official publication of the International Society for AIDS Education.* 2009;21(3):181-206.

**67.** Campbell C, MacPhail C. Peer education, gender and the development of critical consciousness: participatory HIV prevention by South African youth. *Soc Sci Med.* Jul 2002;55(2):331-345.

**68.** B S, T O, T R, et al. HIV Prevention in Yaroslavl, Russia: A Peer-Driven Intervention and Needle Exchange. *Journal of drug issues.* 1999;29(4):777-803.

**69.** Yang Y, Yang K. "Personal Experience, Individual Traits and Attitude Formation: Evidence from China¡¯s Health Service". *Academy of Management Proceedings.* January 1, 2014 2014;2014(1).

**70.** Tang CH, Liu JT, Chang CW, Chang WY. Willingness to pay for drug abuse treatment: results from a contingent valuation study in Taiwan. *Health policy (Amsterdam, Netherlands).* Jul 2007;82(2):251-262.

**71.** Cross MJ, March LM, Lapsley HM, et al. Determinants of willingness to pay for hip and knee joint replacement surgery for osteoarthritis. *Rheumatology (Oxford, England).* Nov 2000;39(11):1242-1248.

**72.** Narbro K, Sjostrom L. Willingness to pay for obesity treatment. *International journal of technology assessment in health care.* Winter 2000;16(1):50-59.

**73.** Rome A, Persson U, Ekdahl C, Gard G. Willingness to pay for health improvements of physical activity on prescription. *Scandinavian journal of public health.* Mar 2010;38(2):151-159.

**74.** Uzochukwu B, Uguru N, Ezeoke U, Onwujekwe O, Sibeudu T. Voluntary counseling and testing (VCT) for HIV/AIDS: a study of the knowledge, awareness and willingness to pay for VCT among students in tertiary institutions in Enugu State Nigeria. *Health policy (Amsterdam, Netherlands).* Mar 2011;99(3):277-284.

**75.** Rosenberger RS, Needham MD, Morzillo AT, Moehrke C. Attitudes, willingness to pay, and stated values for recreation use fees at an urban proximate forest. *Journal of Forest Economics.* 12// 2012;18(4):271-281.

**76.** Martin-Fernandez J, Polentinos-Castro E, del Cura-Gonzalez MI, et al. Willingness to pay for a quality-adjusted life year: an evaluation of attitudes towards risk and preferences. *BMC health services research.* 2014;14:287.

**77.** Salam MA, Noguchi T, Alim MA. Factors affecting participating farmers' willingness-to-pay for the Tree Farming Fund: a study in a participatory forest in Bangladesh. *Environmental monitoring and assessment.* Jul 2006;118(1-3):165-178.

**78.** Lang HC. Willingness to pay for lung cancer treatment. *Value in health : the journal of the International Society for Pharmacoeconomics and Outcomes Research.* Sep-Oct 2010;13(6):743-749.
